# Computational Fluid Dynamics of Intraocular Silicone Oil Tamponade

**DOI:** 10.1167/tvst.10.8.22

**Published:** 2021-07-27

**Authors:** Tommaso Rossi, Giorgio Querzoli, Maria Grazia Badas, Federico Angius, Serena Telani, Guido Ripandelli

**Affiliations:** 1 IRCCS Policlinico San Martino, Genoa, Italy; 2 DICAAR, Università degli studi di Cagliari, Cagliari, Italy; 3 IRCCS Fondazione G.B. Bietti ONLUS

**Keywords:** pars plana vitrectomy, retinal detachment, computational model, computational fluid dynamics, tamponade, shear rate, shear stress, silicone oil

## Abstract

**Purpose:**

To investigate the behavior of silicone oil (SiO) at the steady equilibrium and during saccades and calculate SiO-retina contact, shear stress (SS), and shear rate (SR).

**Methods:**

A 24 mm phakic eye mesh model underwent 50°/0.137s saccade. The vitreous chamber compartment was divided into superior and inferior 180° sectors: lens, pre-equator, postequator, and macula. SiO-retina contact was evaluated as a function of fill percentages between 80% and 90% for a standing patient, 45° upward gaze, and supine. SS and SR for 1000 mPa-s (SiO1000) and 5000 mPa-s (SiO5000) silicon oil were calculated.

**Results:**

SiO fill between 80% to 90% allowed 55% to 78% retinal contact. The superior retina always kept better contact with SiO, regardless of the fill percentage (*P* < 0.01). SiO interface thoroughly contacted the macula only in standing position. SS followed a bimodal behavior and was always significantly higher for SiO5000 compared to SiO1000 (*P* < 0.01) throughout the saccade. The macula suffered the highest mean SS in standing position, while throughout the saccade the average SS was maximum at the SiO-aqueous interface. SR was significantly higher for SiO1000 compared to SiO5000 (*P* < 0.001).

**Conclusions:**

SS on the retinal surface may instantaneously exceed reported retinal adhesiveness values especially at the SiO-aqueous interface and possibly favor redetachment. Despite 90% SiO fill the inferior retina remains extremely difficult to tamponade.

**Translational Relevance:**

Accurate assessment of retina-tamponade interaction may explain recurrent inferior retinal redetachment, silicone oil emulsification, and help to develop better vitreous substitutes.

## Introduction

Pars plana vitrectomy (PPV) is a common surgical procedure for the treatment of retinal detachment that relies on the removal of vitreous gel and the injection of gases or liquids as tamponades. The anatomical success rate of the retinal detachment (RD) surgery currently exceeds 90%.^1^^–^^5^ However, the functional results seem to lag behind.^6^ The reason may lie in the lack of satisfying temporary and long-term vitreous substitutes.^7^ Although Ohm injected the first intraocular gas bubble in 1911,^8^ and Cibis pioneered the use of the silicone oil (SiO) in 1962,^9^ the perfect vitreous substitute has yet to be found.

The gas and fluid bubbles introduced into the eye interact with the retina and vitreous chamber boundaries both statically and dynamically, in a complex manner, as ocular saccades determine angular acceleration exceeding 10,000 degrees per square second.^10^

Computational fluid dynamics (CFD) modeling allows the accurate calculation of physical parameters that are impossible to estimate in vivo and can be applied to several fields in medicine, including ophthalmology.^11^ A few studies applied CFD to predict the equilibrium shape of the aqueous-tamponade interface. However, none of them analyzed the shear stress distribution and the percentage of retinal surface in contact with SiO.

Isakova et al.^12^ studied the case of two tamponades (gas and silicone oil) with different filling ratios, while Pralits et al.^13^ investigated endothelial graft coverage in the case of Descemet's membrane endothelial keratoplasty. Finally, Angunawela et al.^14^ evaluated the shear stress on the retina over the surface of an idealized spherical eye geometry.

The purpose of the present study is using the CFD to investigate the behavior of silicone oil at the steady equilibrium and during ocular saccades to calculate the SiO-retinal surface contact and the shear rate over the various regions with different patient postures and fill fractions. The surgical and clinical relevance of such parameters is significant since shear stress may favor redetachment and interfere with retinal functioning while the shear rate is felt to be an important factor responsible for SiO emulsification.

## Materials and Methods

### CFD Model

The simulations were performed by integrating the equations of fluid mechanics by means of the open-source library OpenFOAM, which is based on the finite volume method and was previously validated in a large number of engineering and biomedical applications, including the eye.^15^

**Figure 1.**
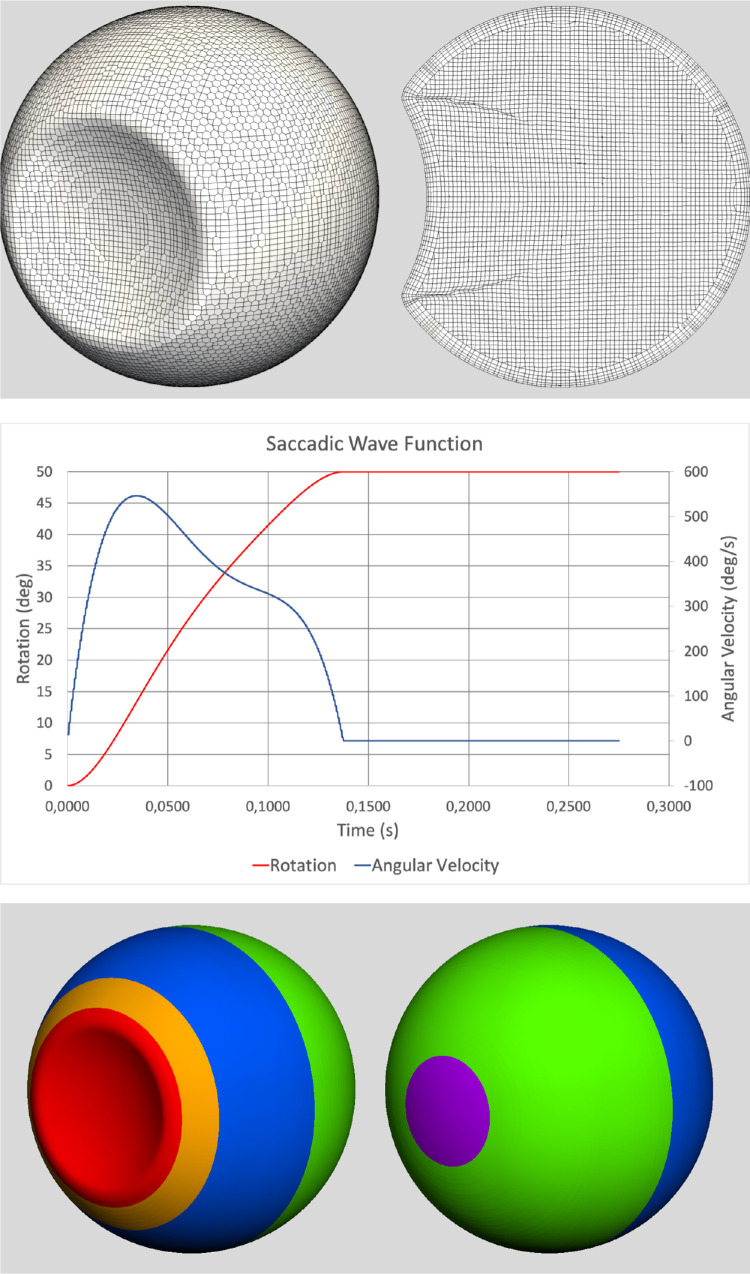
a) Mesh geometry b) Saccade angle of rotation (*red line*) and angular velocity (*blue line*) c) Vitreous chamber surface segmentation: anterior and posterior view. *Purple* represents the macula, *green* the postequatorial retina, *blue* the preequatorial retina, *orange* is the ora serrata and *red* is the lens posterior surface.

We here describe the adopted procedure in general, while more details on the numerical methods are given in the Appendix. The vitreous chamber was modeled as a spherical mesh (24 mm in diameter), with a spherical cap on the anterior side mimicking the indentation of the lens as described elsewhere.^11^^,^^15^ A view of the body conformal hex-dominant mesh adopted for the simulations is displayed in Figure 1a.

The vitreous chamber was ideally filled with SiO (Polydimethilsyloxane (PDMS) 1000 mPa-s or 5000 mPa-s dynamic viscosity, hereafter referred to as SiO1000 and SiO5000, respectively) and aqueous in different volumetric ratios. Though tamponade agents are generally considered viscoelastic fluids, the elastic component is relevant, with respect to a viscous one, only in the case of high molecular weight (HMW) polymers addition.^16^^,^^17^ We considered only SiOs composed by 100% PDMS, both SiOs and aqueous were modeled as Newtonian fluids.

The flow equations were solved numerically, using the volume of fluid (VOF) technique ^18^ to model the fluidics of both the silicone oil and water fraction. No-slip conditions were assumed at the eye wall. As a consequence, the fluid adjacent to the eye wall adheres to the moving surface, while the inner fluid layers are free to flow over each other obeying to the Newtonian constitutive law.

The effect of the surface tension was modeled by imposing a variable contact angle at the line of water-oil-retina interface. While static contact angles for tamponades are explicitly documented in the literature,^19^ the receding and advancing angles were inferred from the experimental work by Chan et al.^20^ and are reported in Table 1.

**Table 1.** Physical Properties of Involved Fluids

	SiO 1000	SiO 5000	Aqueous
Density	980 kg/m^3^	980 kg/m^3^	1000 kg/m^3^
Dynamic viscosity	1000 mPa-s	5000 mPa-s	1 mPa-s
Interfacial tension vs. water	0.044 N/m	0.044 N/m	
Static contact angle vs. water and retina	16.2°	16.2°	
Dynamic contact angle (advancing)	21.2°	21.2°	
Dynamic contact angle (receding)	11.2°	11.2°	

Three patient's positions were considered: standing, 45° upward gaze, and supine with SiO1000 and SiO5000 fill ranging from 80% to 100%, at 5% increments. For each simulation, the tamponade interface was initially set horizontal, then the model was run until static equilibrium was reached. As expected, the SiO rises to the top of the eye and the aqueous compartment settles underneath. The equilibrium shape is affected by the interfacial tension and by gravity, but not by viscosity. The same static equilibrium condition is taken as initial state for the dynamic simulations of the two considered SiO tamponades.

Starting from the static equilibrium, a saccade was imposed on the vitreous chamber for each of the 30 combinations of SiO tamponade, filling volume and patient posture were considered. In contrast to the static equilibrium, the viscosity of SiO affects the dynamic behavior, and the motion of SiO1000 and SiO5000 during the saccades differs. Different simulations were run starting from the same static equilibrium condition. A rotation amplitude of 50° was imposed to the eye, with a duration of 0.137 seconds (Fig. 1b), attaining a peak angular velocity of 547° per second, 0.034 seconds after the beginning of the rotation. Following Stocchino et al.^21^ a realistic saccadic function was built using a polynomial function:

$$\begin{array}{c} \theta \left(t\right)=c_{0}+c_{1}t+c_{2}t^{2}+c_{3}t^{3}+c_{4}t^{4}+c_{5}t^{5} \end{array}$$

where t represents the time, θ the angular displacement, and the coefficients c_1_,.., c_4_ are summarized in Table 2. The angle of rotation and angular velocity are plotted versus time in Figure 1b. After the maximum rotation was achieved, the simulation was run for 0.137 additional seconds, to observe the fluid deceleration following the saccade. The fifth order polynomial ensures that acceleration is continuous and varies continuously in time.

**Table 2.** Saccadic Wave Function Coefficients

c_0_[°]	c_1_ [°/s]	c_2_ [°/s^2^]	c_3_ [°/s^3^]	c_4_ [°/s^4^]
0	0	2.01 × 10^4^	−3.29 × 10^5^	2.30 × 10^6^

### Retinal Surface Segmentation

The eye CFD model was divided into the topographic regions shown in Figure. 1c:

- Macula: the retinal surface centered on the geometric posterior pole and extending 20° anteriorly
- Postequatorial retina: the surface posterior to the geometric equator and comprised between the equator and the macula as above delimited
- Anterior retina: the surface comprised anteriorly between the geometrical equator and the pars plana.

All the above regions have been further divided into superior and inferior 180°.

### Main Outcome Measures

• Instantaneous maximum shear stress (IMSS): maximum wall shear stress over the investigated region computed at each instant (measure unit: Pa)
• Pointwise maximum shear stress (PMSS): maximum value of the wall shear stress over the time, computed at each location of the eye wall (measure unit: Pa)
• Vertical average shear stress (VASS): vertical component of the wall shear stress averaged at each location and over the simulation time (measure unit: Pa)
• Average regional shear stress (ARSS): instantaneous magnitude of the wall shear stress spatially averaged over the region of interest at each time of the simulation (measure unit: Pa)
• Instantaneous maximum shear rate (IMSR): maximum wall shear rate over the investigated region, computed at each instant (measure unit: 1/s)

### Statistical Analysis

Statistical analysis used analysis of variance (ANOVA) to evaluate the significance of shear stress values at different locations and to compare different regions depicted in Figure 1c. For all the analyses, *P* values less than 0.05 were considered statistically significant.

## Results

### Silicone Oil-Retinal Surface Contact

**Figure 2.**
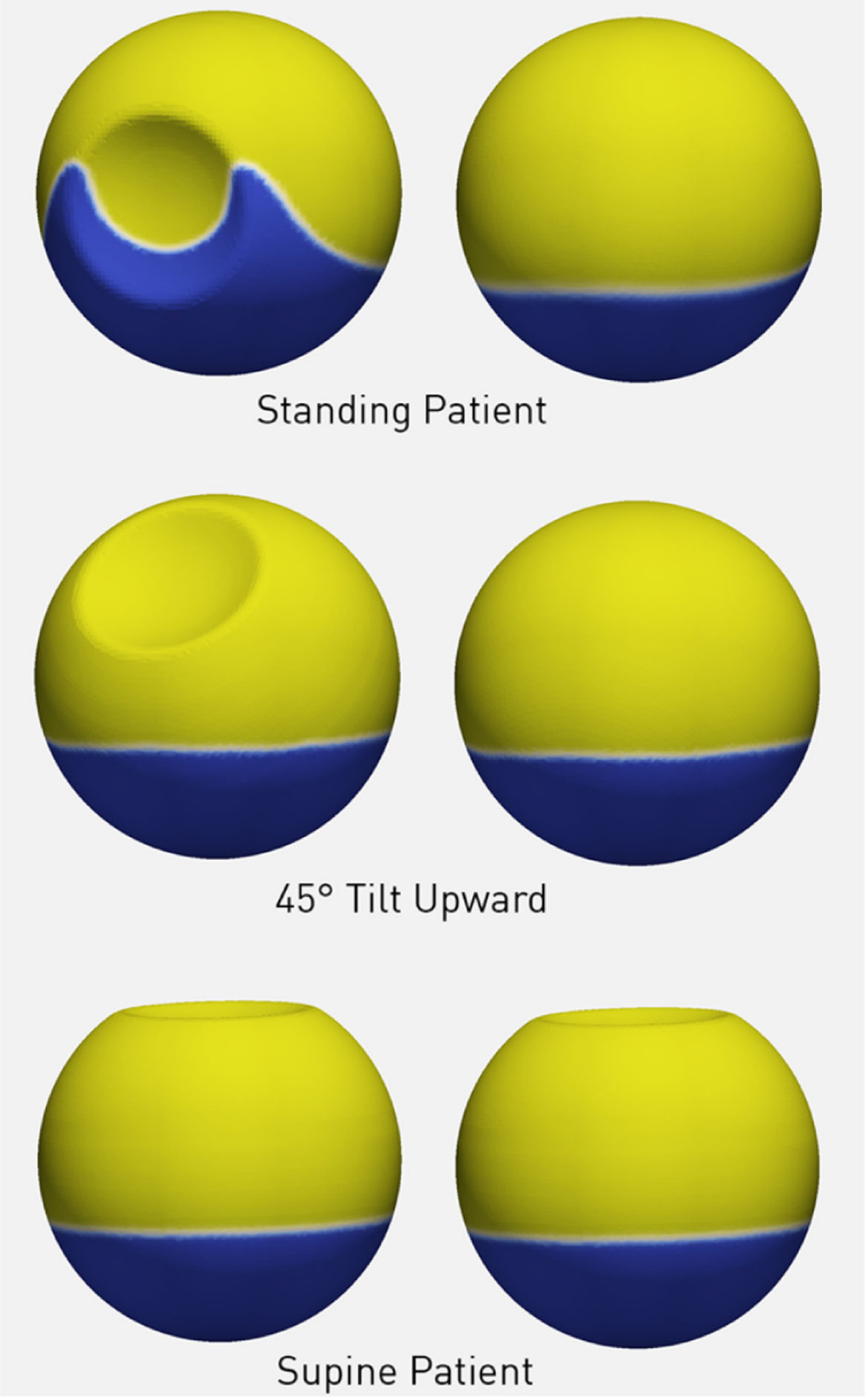
3D view of vitreous chamber: anterior (*left row*) and posterior (*right row*) view. Silicone oil is represented in *yellow* and aqueous in *blue* for 90% tamponade fill. Note that in a standing patient (*upper left image*) the aqueous fills the recess between the lens and the ora serrata raising (the aqueous) to a much higher level than anywhere else.

Figure 2 shows a three-dimensional view of the static equilibrium for 90% fill percentage for the analyzed patient positions. SiO floats on aqueous, but, only in the case of a standing patient, the aqueous level between the lens and anterior vitreous rises above the lower meniscus level at the posterior pole due to capillary action (Fig. 2a). The behavior agrees with what previously observed in silico with a similar eye geometry.^12^

**Figure 3.**
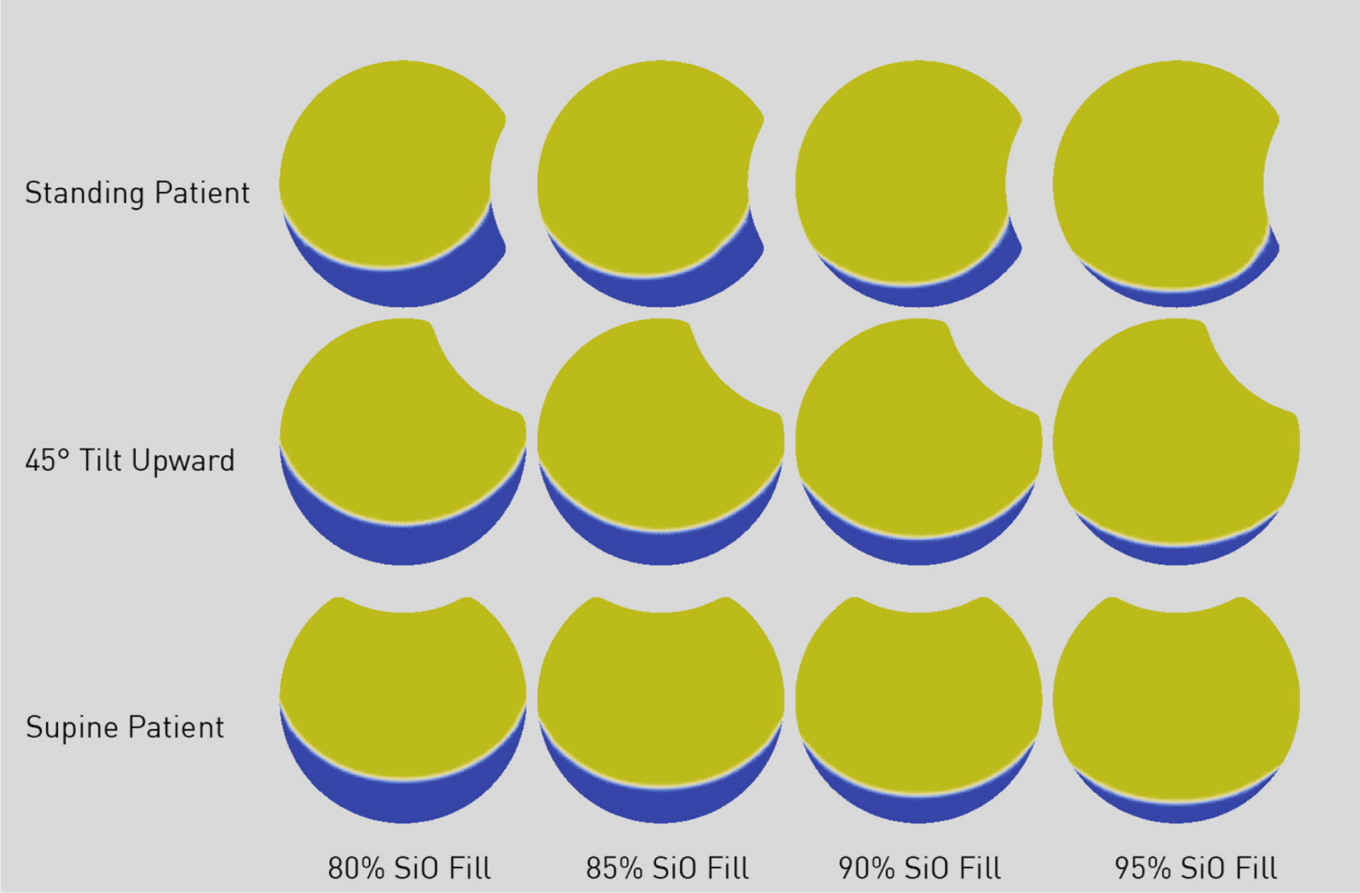
2D view of silicone oil bubble shape as a function of fill percentage (columns) and patient position (rows). Silicone oil is represented in *yellow* and aqueous in *blue*. Note that less than 90% fill leave the inferior pre- and postequatorial retina almost entirely in contact with aqueous. (See also Figs. 4 and 5).

**Figure 4.**
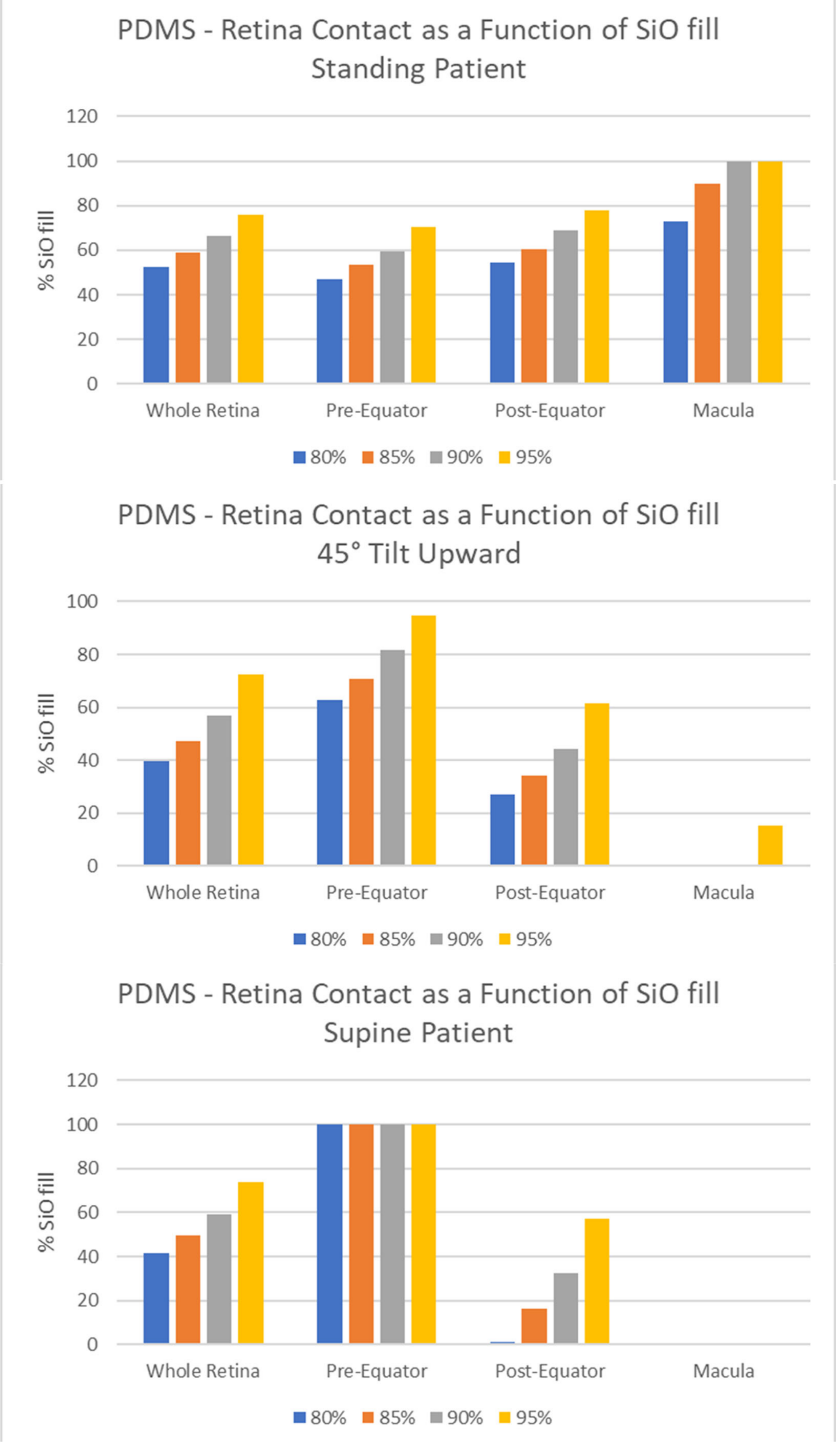
Silicone oil-retina contact (percentage of SiO-wetted retinal surface), bar chart for a) standing patient; b) 45° head tilt upward; and c) supine patient.

The amount of retinal surface in contact with SiO as a function of the vitreous chamber fill percentage (ranging from 80% to 95%) is shown in Figure 3 and analyzed in Figure 4 for the retina, macula, and pre- and postequatorial sectors. Different head positions returned significant difference in values for retina-SiO contact at the macula, and pre- and postequatorial retinal sectors (compare Figs. 4a, 4b, and 4c).

**Figure 5.**
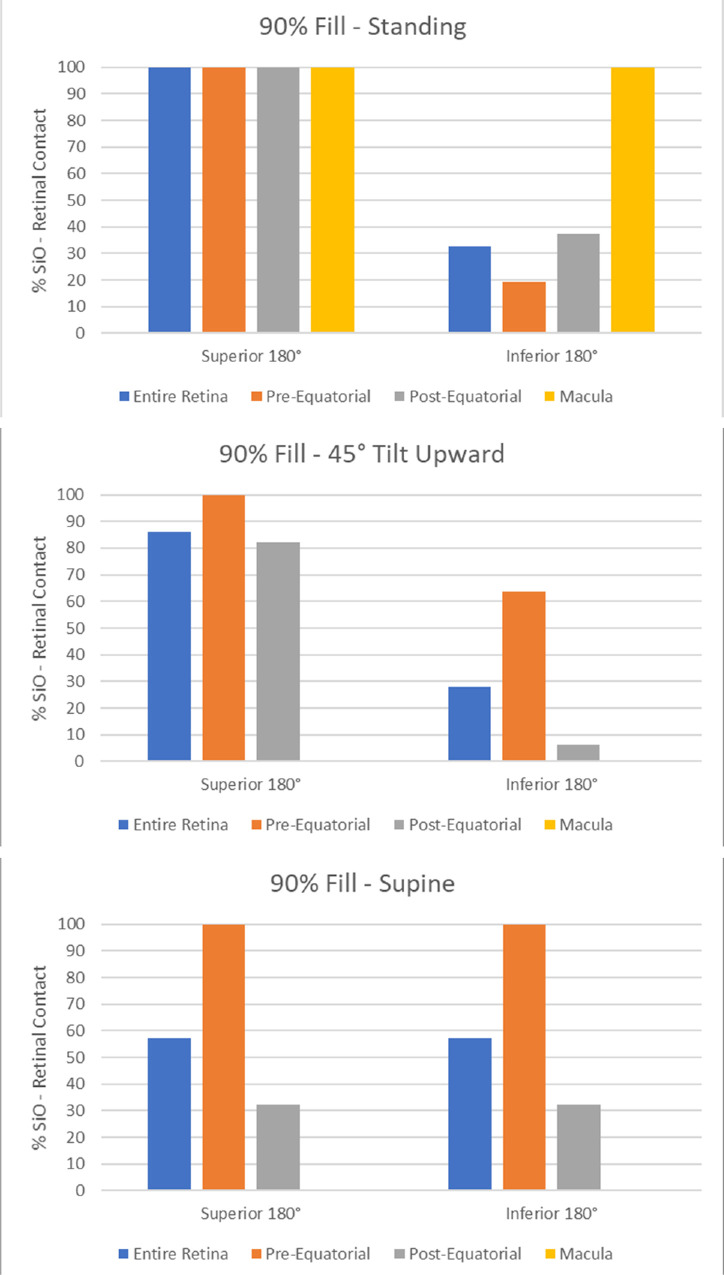
Silicone oil-retina contact (percentage of SiO-wetted retinal surface). Data referred to the superior and inferior retina have been displayed separately in this bar chart for a) standing; b) 45° head tilt upward; and c) supine patient.

Since the SiO floats on aqueous, the percentage of superior retina in contact with SiO is significantly higher compared to inferior retina for a standing and 45° upward gazing patient (Figs. 5a and 5b, respectively): a 90% SiO fill contacts only about 30% of the inferior hemiretina and less than 20% of the pre-equatorial inferior sector. The upward gaze and supine position leave the entire posterior pole completely out of contact with SiO (Figs. 5b and 5c).

In the supine position the contact line is confined to the spherical portion of the eye chamber, and an analytical computation of the retina coverage fraction was performed following Eames et al.^19^ For the considered fill percentages, the comparison between the theoretical and CFD-derived retina surfaces is acceptable, with differences ranging from 1.8% to 4.8%.

### Shear Stress

The instantaneous maximum shear stress (IMSS) calculated at the retinal surface as a function of time follows a bimodal behavior (Fig. 6), peaking at the beginning and end of ocular rotation, when rotational acceleration is maximum. Maximum shear stress over the whole simulation time, shown in Figure 7 for the standing patients, is significantly higher for SiO5000 compared to SiO1000 at any considered fill percentage (*P* < 0.01) and throughout the saccade (compare Figs. 6a to 6b).

**Figure 6.**
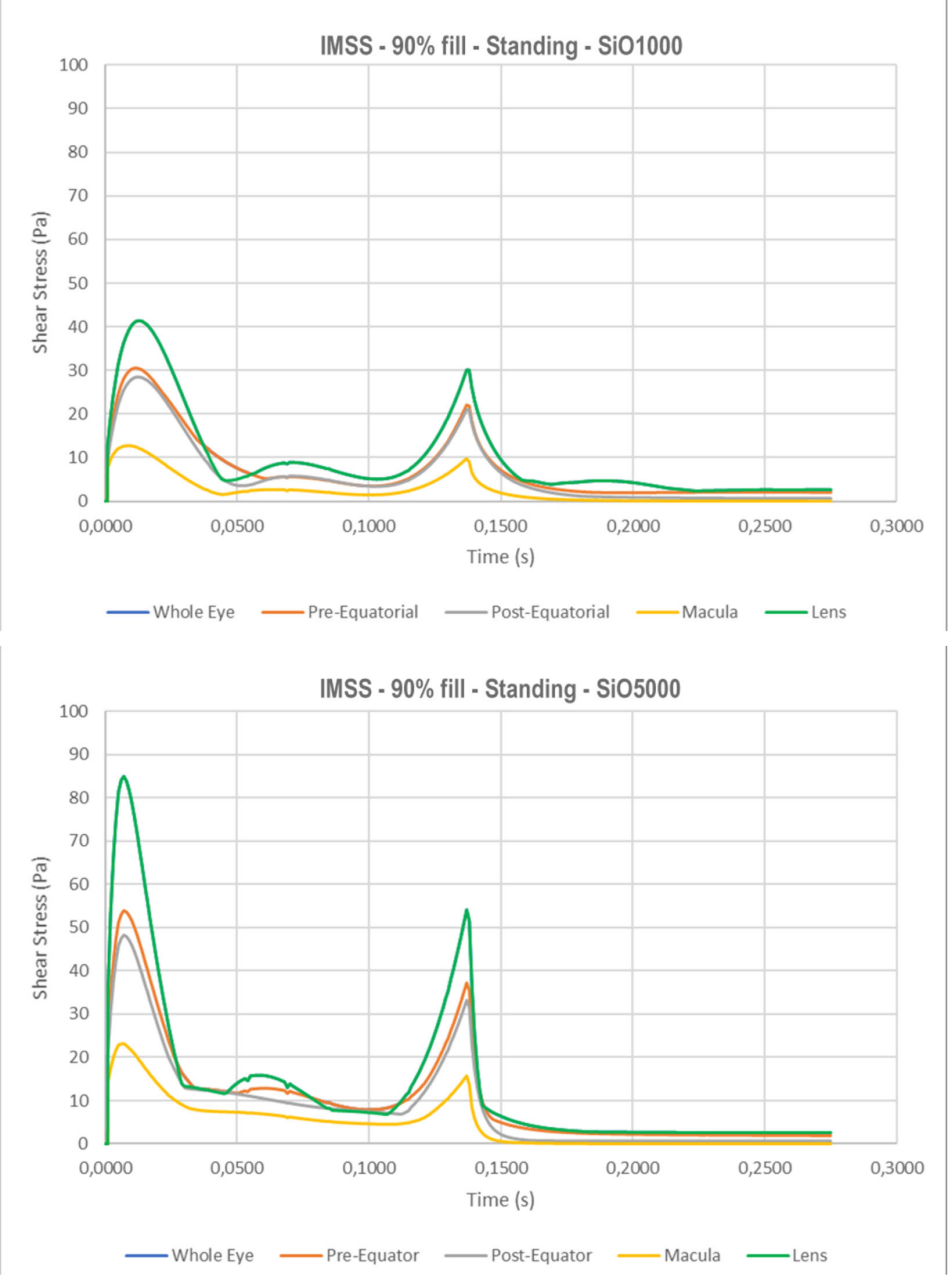
Instantaneous maximum shear stress as a function of time throughout the saccade. Note a bimodal behavior with a first peak when motion starts and a second when the eye suddenly stops while silicone oil keeps moving. The lens is exposed to the highest maximum shear stress while the macula faces the lowest values irrespective of patient position: a) standing with SiO1000; b) standing with SiO5000; c) 45° head tilt upward with SiO1000; and d) supine with SiO1000.

**Figure 6.**
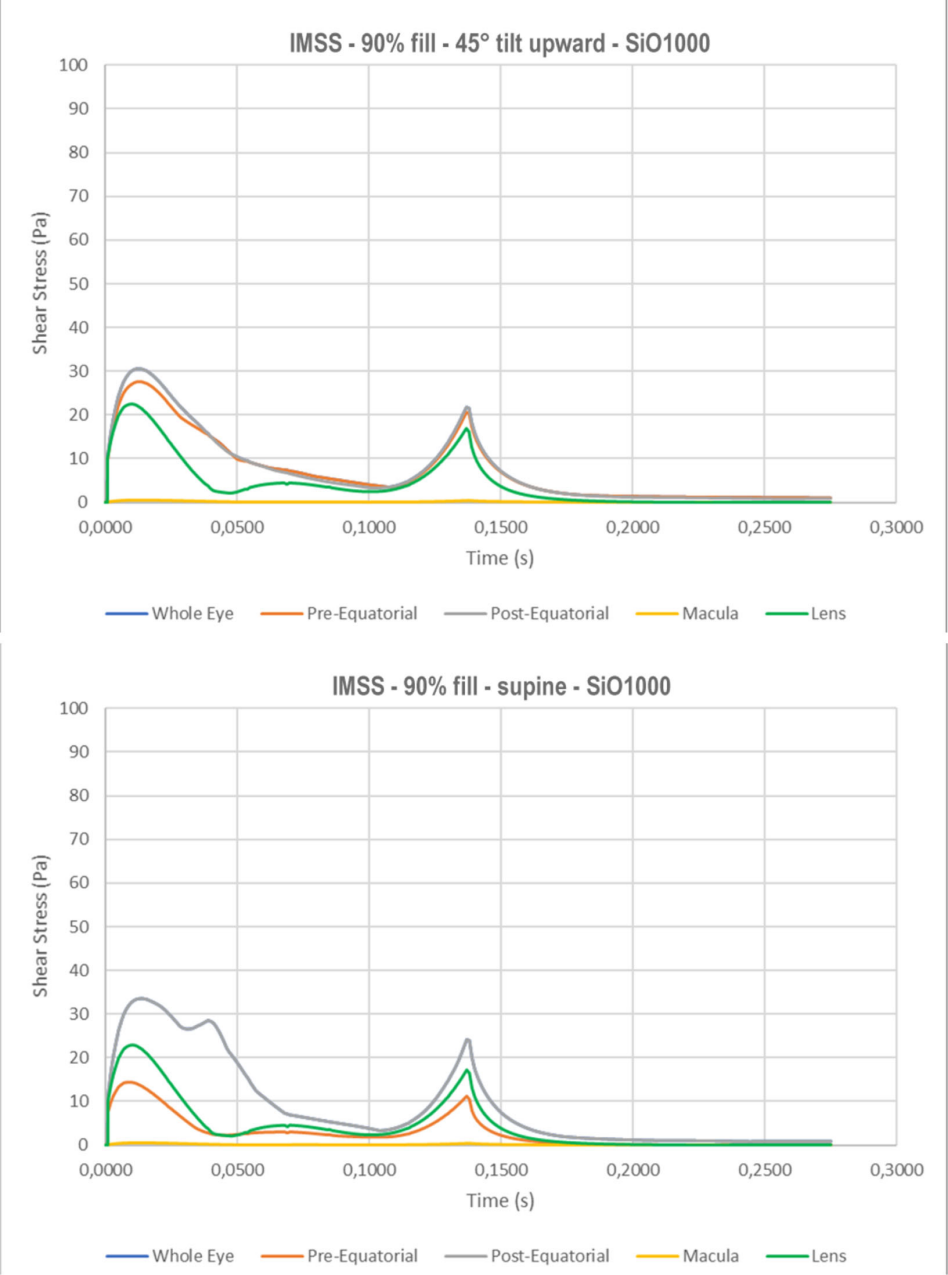
Continued.

**Figure 7.**
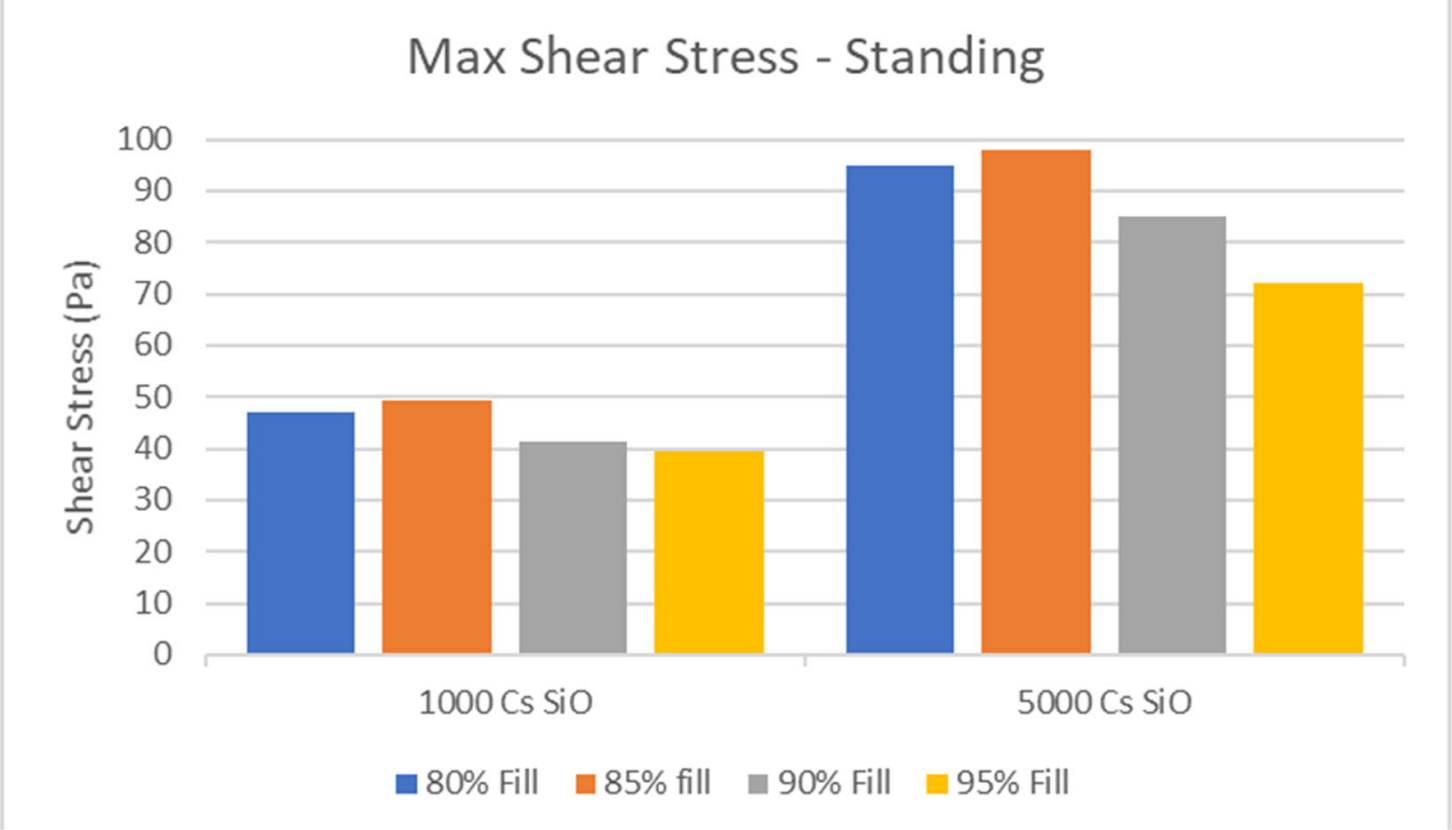
Maximum shear stress calculated in retinal sectors as a function of fill percentage with SiO1000 and SiO5000. Note that higher viscosity determines a significantly higher shear stress regardless to fill rate.

The highest IMSS for a standing patient occurs at the lens, followed by the pre- and postequatorial retina, while it is significantly lower at the macula (Fig. 6a). As the eye changes position to 45° upward gaze (Fig. 6c) and supine (90°; Fig. 6d), IMSS at the lens and macula significantly reduces while the pre- and postequatorial retina remains similar.

**Figure 8.**
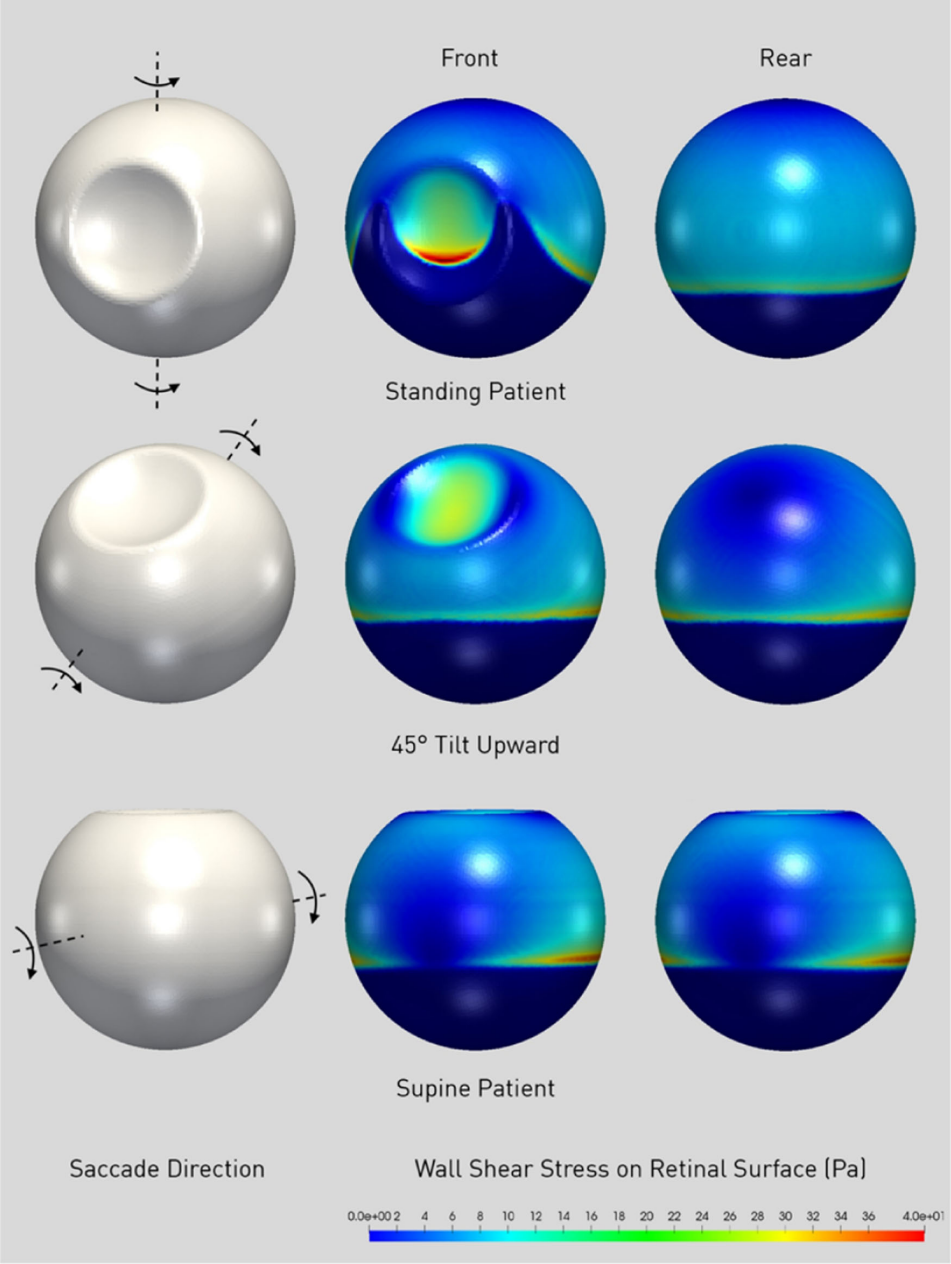
3D maps of the pointwise maximum shear stress (PMSS) for a 90% SiO1000 fill during saccadic rotation as indicated in the *grey phantoms* to the left. The *central column* is a front view while the *right column* is a posterior view. Note how shear stress is negligible where aqueous touches the retina but is a maximum at the interface with silicone oil as well as on the sides of the eye where the tangential velocity is nearly orthogonal to the contact line.

Independent of the head orientation, the three-dimensional rendering of pointwise maximum shear stress (PMSS) throughout the saccade (Fig. 8) shows high values behind the crystalline lens and along the whole SiO-aqueous meniscus. The highest values at the meniscus are found where it tends to be orthogonal to the rotational velocity of the wall, that is, on the sides of the anterior chamber.

**Figure 9.**
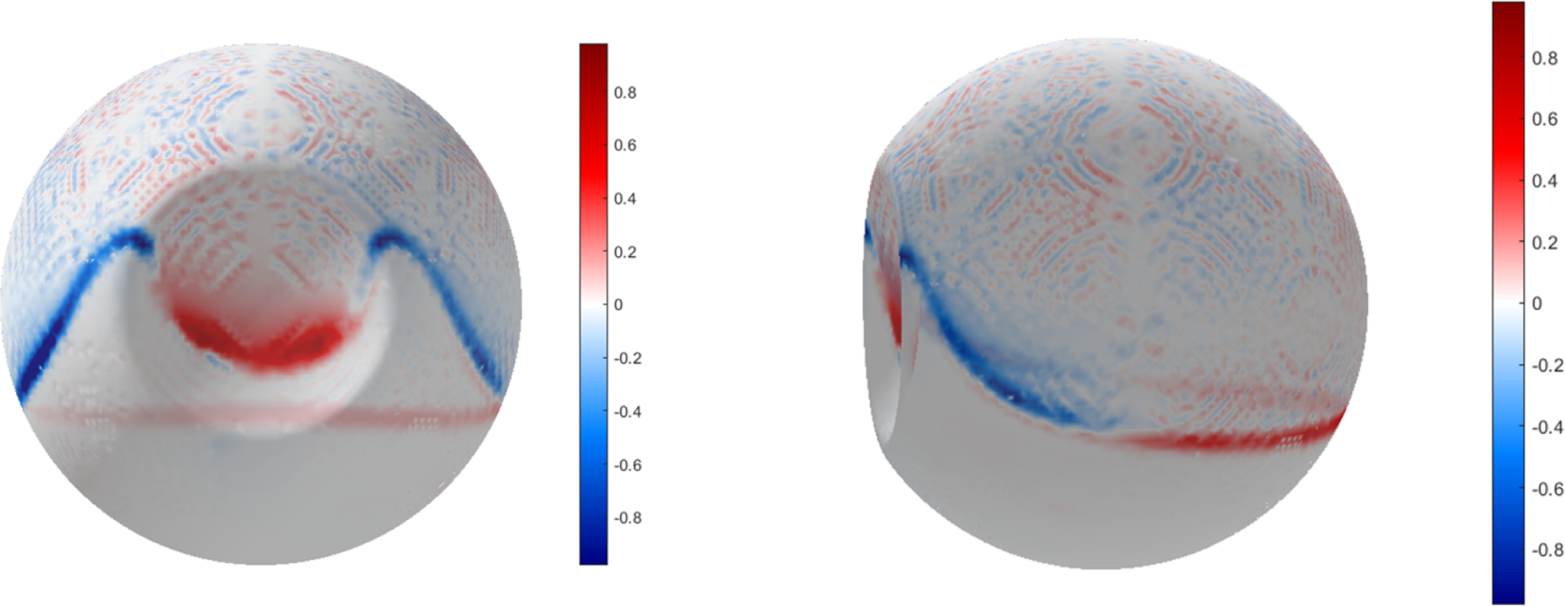
Vertical average shear stress (VASS) 3D maps. Anterior view and side view are shown in *left* and *right panel*, respectively. *Red* color indicates that the shear stress points upward while *blue* indicates that the shear stress points downward. Note that resulting shear stress vector is almost null throughout the surface except at the silicone oil-retina interface and points superiorly over all the postequatorial retina (and the lens) and inferiorly over the pre-equatorial retina.

The time average of the vertical component of the shear stress vector (VASS) for a standing patient was calculated and reported in supplemental digital content (SDC) Figure 9: the net resulting vector points upward at the macula (red region in the map) and downward in the pre-equatorial area (blue region in the map).

The highest regional mean shear stress, ARSS, in a standing patient occurs at the macula (Fig. 10a with SiO1000) and increases significantly with SiO5000 (Fig. 10b); 45° tilt and supine positions resulted in higher ARSS at the pre- and postequatorial retina but negligible at the macula (Figs. 10c, d).

**Figure 10.**
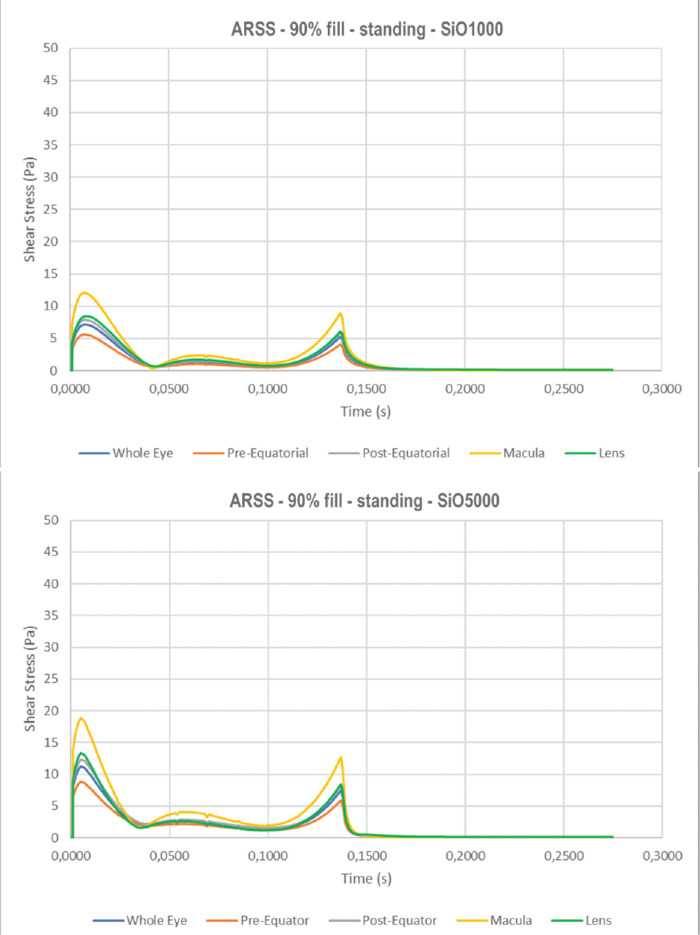
Average regional shear stress (ARSS) as a function of time for 90% tamponade fill. The macula is exposed to the highest mean shear stress throughout the saccade while all other sectors show significantly lower peak values: a) standing with SiO1000; b) standing with SiO5000; c) 45° tilt upward head with SiO1000; and d) supine with SiO1000.

**Figure 10.**
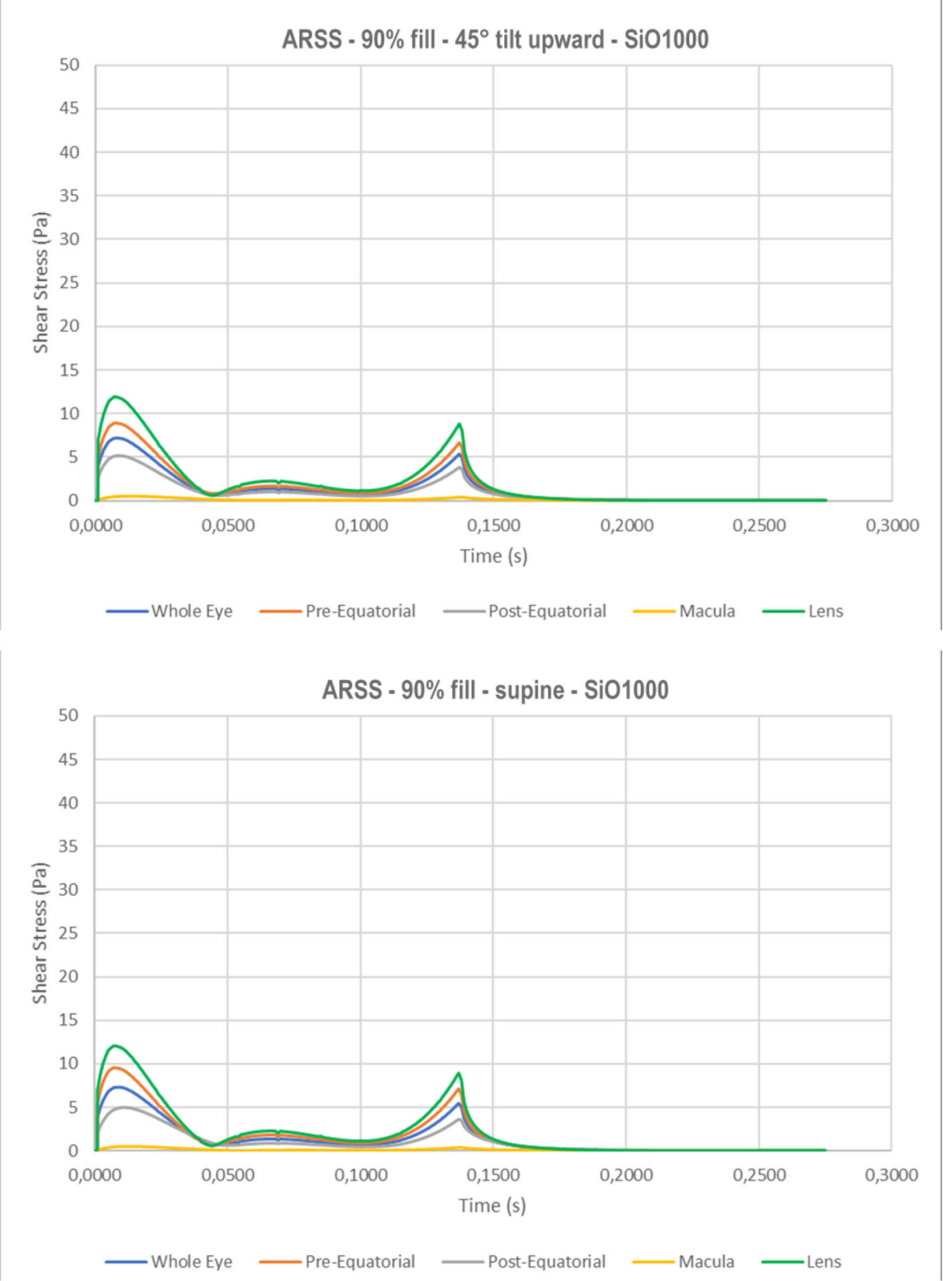
Continued.

### Shear Rate

**Figure 11.**
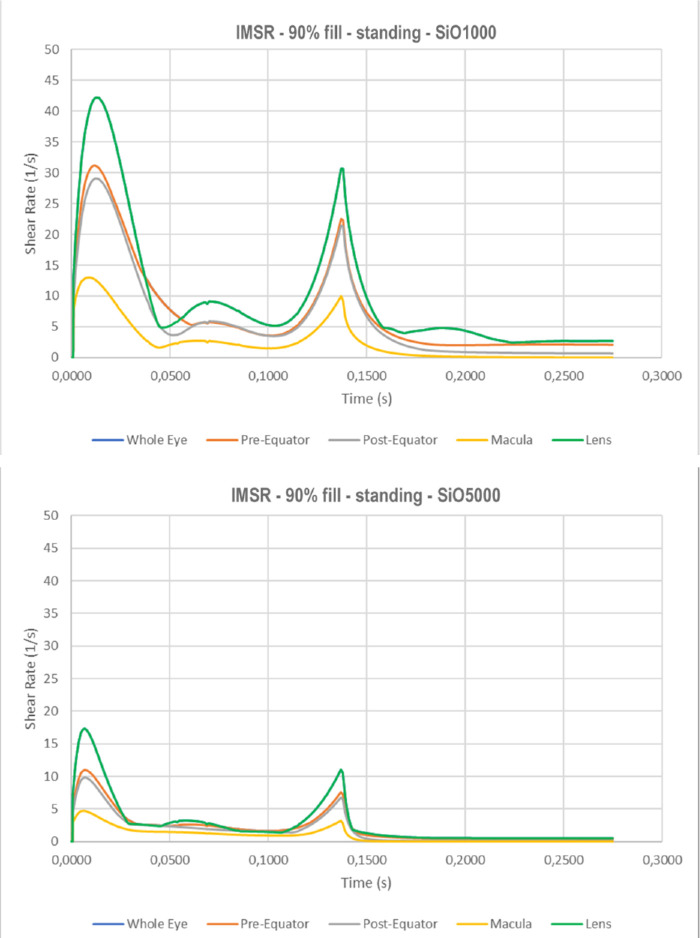
Instantaneous maximum shear rate for a standing patient as a function of time in case of 90% tamponade fill. a) SiO1000; and b) SiO5000.

The instantaneous maximum shear rate (IMSR) curve exhibits bimodal distribution (Fig. 11), similar to IMSS (see Fig. 6), except SiO1000 shows a significantly higher (double) shear rate compared to SiO5000 (Figs. 11a and 11b) since, for a Newtonian fluid, the shear rate equals shear stress/2τ (τ = viscosity). Therefore, the highest viscosity, SiO5000 (Fig. 10b), shows lower shear rates than SiO1000 (Fig. 10a) at any location (*P* < 0.01).

## Discussion

The ideal physical and chemical properties of tamponades are far from being identified and even their mechanisms of action are not yet completely understood.^22^ The term “tamponade,” nowadays, probably best pertains to the capacity of “covering” retinal tears, preventing aqueous leakage through them. Buoyancy, once believed to be the main player, plays a minor role compared to superficial tension and viscosity, indeed the ability to displace aqueous from the retinal tear(s) has been suggested to be the single most important factor.^23^

To exert their beneficial effects tamponades must be in contact with the retinal tears during the ocular motion and in the different patient postures. This is why the amount of retina in contact with SiO is a key factor for retinal reattachment and durable success as most RDs recur inferiorly when lighter-than-water tamponades are used and superiorly with heavier-than-water ones.

While the few studies dealing with the equilibrium shape of the aqueous-tamponade interface present their results in terms of overall contact with idealized or realistic eye shapes,^12^ we computed the fraction of retina (not just the fraction of the whole vitreous chamber surface) in contact with the tamponade, which is more clinically relevant. Our model confirmed that the retinal surface covered by SiO is highly dependent on fill fraction: even at 90% volume fill, more than 30% (Fig. 4) of the retinal surface is still untouched by SiO, increasing the risk of recurrent RD. Due to buoyancy, the inferior retina is hardly in contact with SiO whenever the patient stands and entirely in aqueous when looking up (Fig. 5). Even the macula is only partially covered by SiO in a standing patient unless a 90% fill is warranted (Fig. 4a).

Interestingly, the interfacial tension prevents SiO from adequately entering the recess between the lens and the ciliary body when the patient is in standing position, allowing aqueous to raise higher than the meniscus at the retinal surface (Fig. 2), an observation already made by Williams and Wong.^24^ around the indentation caused by the presence of buckling elements.

Shear stress occurs whenever a force is applied tangential to a surface. Saccades create shear stress on the retina with a typical bimodal behavior over time (Fig. 6). The peaks correspond to the phases of maximum angular acceleration and deceleration of the eye: as motion starts, the SiO bubble lags, creating friction against the moving retina, while after the eyeball stops, its momentum maintains inertial motion against the immobile retina (see video S1). The higher viscosity of SiO5000 results in almost twice as much as the shear stress of SiO1000, through the same mechanism and regardless of fill percentage (Fig. 7).

From the clinical standpoint, shear stress changes have not been overtly linked to detrimental retinal effects, but Muller and retinal ganglion cells^25^^,^^26^ modulate gene expression in response to shear stress.^27^ We observed that the *bursa premacularis* drastically reduces shear stress at the fovea^11^ until aging disrupts the physiologic adhesion resulting in the posterior vitreous detachment, which in turn is associated with inflammation and progression of age-related macular degeneration.^28^

It should also be noted that, for a standing patient, the macula is exposed to the lowest maximum shear stress (IMSS) but the highest mean shear stress (ARSS) (Fig. 8). The shear stress increases with tamponade viscosity since that region is entirely in contact with SiO and subject to the highest tangential velocity, being at the highest distance from axis of rotation. This means a sustained stress at the macula throughout ocular movement that increases with tamponade viscosity.

Although surface roughness at the molecular level and the purported presence of a thin aqueous layer^14^^,^^29^ may act as confounders, our model calculated shear stress peak above 80 Pa over the lens and 50 Pa over the retina for SiO5000 and 40 Pa and 30 Pa, respectively, for SiO1000 (Fig. 6). Such values are significantly higher than vitreous shear stress at the fovea previously published by Rossi et al.^11^ who calculated approximately 5 Pa in the presence of the bursa premacularis and 10 Pa after complete posterior vitreous detachment.

It should be noticed that the retinal detachment or reattachment is related to the forces directed orthogonally to the retinal surface as discussed, for example, in Foster and Chou,^30^ Foster et al.,^31^ and Chou and Siegel,^32^ while the shear stresses are oriented tangentially. However, the shear stresses play a complex and indirect mechanical role in the process by promoting the on-plane displacement of the retina with respect to the retinal pigment epithelium, thus weakening cellular adhesion.

Additionally, since most retinal tears occur at the equator or anterior to it, it is conceivable that saccade induced shear stress may intermittently lift a retinal tear flap or at least reduce its adhesion and allow aqueous fluid to enter the subretinal space whenever changes in ocular and head position bring the SiO-aqueous meniscus in proximity to retinal tears. Head and/or eye motion can also result in a sudden shear stress change as the SiO-aqueous meniscus spans the retinal break. Quantification of these effects, however, involve a detailed modeling of the fluid-structure interaction that is out of the scope of the present work.

The shear stress may be responsible for the retinal displacements observed after RD surgery as the temporal retina shifts slightly inferiorly with SiO tamponade and superiorly when gas is introduced in the vitreous chamber.^33^^,^^34^ We observed that the net resulting shear stress vector along the vertical axis throughout the saccade (Fig. 9) points upward posteriorly to the equator and downward anteriorly, thus generating globally a downward movement on the retina. This may explain the puzzling observation of retinal displacement after RD surgery with SiO tamponade.^33^ It should be noted that although we computed a single saccade in one direction, the opposite saccade in a standing patient, would null any resulting vector component lying on the horizontal plane (because of the symmetry of the phenomenon) and add an identical vertical vector with the same direction along the vertical (z) axis, thus nearly doubling its module and the resulting tilting movement on the retina. The resulting movement may cause the observed retinal displacement, especially in the first hours after surgery when residual subretinal fluid remnants prevent formation of the chorioretinal adhesion, although biologic processes leading to increased retinal stiffness may well also participate.

The maximum shear rate during the saccade obviously shows similar overall behavior to shear stress. Compared to SiO1000, higher viscosity tamponade, SiO5000, produces slightly higher shear stresses and significantly lower shear. That behavior is not surprising as shear rate equals shear stress/2τ (where τ indicates viscosity). Therefore, increasing the viscosity, a given shear stress corresponds to a lower shear rate and, conversely, a given shear rate corresponds to a higher shear stress.

Higher viscosity SiO (5000 mPa-s) therefore produces slightly higher shear stress on the retina and may be more challenging to inject through a small gauge instrument but determines a lower shear rate, which is believed to promote emulsification.^20^ Chan et al.^29^ estimated the shear rate, macroscopically measuring SiO angular displacement in a Perspex cylinder, and concluded that an increased viscosity and the presence of an indentation reduced its magnitude. This last result would justify the alteration of vitreous chamber geometry through buckling elements.^15^

Balancing the trade-off of higher and lower viscosity silicone oils is still far from having reached consensus: visual and anatomical results, in fact, fail to show significant difference in most studies: Scott et al.^35^ compared surgical series treated with 5000 and 1000 centistokes (cSt) PDMS and found no statistical difference in redetachment rate, post-op visual acuity, and complication rates.^36^ The higher tendency toward emulsification of lower viscosity PDMS seems to be the only consistent piece of information.^16^

The inferior relapsing of retinal detachment despite SiO tamponade and regardless of viscosity is probably the consequence of different pathogenic mechanisms. Early redetachment in the absence of overt proliferative vitreoretinopathy (PVR) could be related to intermittent failure of the silicone oil bubble to efficiently cover the retinal tear(s), allowing aqueous inflow into the subretinal space, whereas late recurrences usually imply cell proliferation and retinal contraction likely to be favored by inflammation mediators pooling within the inferior aqueous reservoir.

In summary, the application of CFD to silicone-oil-filled eyes during saccadic motion clarifies the fill fraction, the topography of SiO-retina contact, and the shear stress applied to the retinal surface.

We acknowledge the limits of our study mostly related to the uncertainties of retina-silicone oil static and dynamic interactions and the variations of normal human anatomy from the proposed mesh.

## Supplementary Material

Supplement 1

## Appendix Appendix: Computational Methods

We solved the governing equations for a 3D unsteady laminar two-phase flow; namely, the continuity equation, the full momentum balance equations (Navier-Stokes equations), and the transport equation for the water volume fraction. The tamponade/aqueous interface was approximated using the volume of fluid (VOF) approach.^18^ The equations were spatially solved on the computational grid using second-order accurate, unstructured finite-volume schemes to allow for handling of the eye complex geometry. Temporal advancement of the flow and of the volume fraction was performed using the implicit, first-order accurate, Euler scheme. The pressure-implicit-split-operator (PISO) method^37^ was used for the solution of the pressure-velocity coupling.

The fluid domain was discretized with an unstructured mesh consisting of hexahedral and tetrahedral cells, using the grid generation tool snappyHexMesh, included in the OpenFOAM library. The mesh was chosen after performing refinement independence tests to guarantee a solution independent of grid size. The working grid consisted of 350,000 cells and was characterized by five layers of extruded hexahedral cells of progressively increasing height at the eye surface (see Fig. 1a).

For the saccade simulations the dynamic mesh technique was adopted, and the grid was rigidly moved according to the law of motion reproducing the saccade.

## Supplementary Material

Supplementary Video S1. Video shows 3D representation of shear stress magnitude during 50° saccade in standing, upward tilting, and supine position.
